# High-Temperature Resistance of Modified Potassium Magnesium Phosphate Cement

**DOI:** 10.3390/ma15248967

**Published:** 2022-12-15

**Authors:** Yuqi Yang, Yan Liu, Zizhuo Yan, Zhuoyi Chen

**Affiliations:** School of Civil Engineering, Changsha University of Science & Technology, Changsha 410114, China

**Keywords:** potassium magnesium phosphate cement, magnesium to phosphorus mass ratio, mechanical properties, high-temperature performance

## Abstract

To study the high-temperature mechanical properties of potassium magnesium phosphate cement mortar and the high-temperature resistance of its laminates. Potassium magnesium phosphate cement (MKPC) was prepared by using heavy-burning magnesium oxide and potassium dihydrogen phosphate as the main raw materials, borax as the retarder, and compounded with a certain amount of fly ash and silica fume. The effect of the mass ratio of magnesium to phosphorus (M:P), compounded fly ash and silica fume on the setting time and mechanical properties of MKPC was investigated. Furthermore, based on the better M:P, the compressive strength of MKPC mortar was studied after 3 h of constant temperature at 400 °C, 600 °C, and 800 °C, and the effect of fly ash and silica fume on the high-temperature resistance of MKPC was analyzed. The high-temperature resistance of MKPC was further evaluated by analyzing the temperature variation of potassium magnesium phosphate cement laminate during a constant temperature of 650 °C for 3 h. The results showed that the mechanical properties of potassium magnesium phosphate cement were influenced by different raw material ratios, and the mechanical properties of potassium magnesium phosphate cement were optimal when M:P was 2:1, fly ash was 5% and silica fume was 15%. The internal temperature of MKPC laminate increased slowly with time, and its high-temperature resistance was better.

## 1. Introduction

Along with the rapid development of urbanization, building fires have gradually become a major factor threatening the safety of human life and property. The study of the mechanical properties of building materials under high temperatures is of great theoretical and practical significance for the evaluation of structural fire resistance and post-disaster restoration work [1]. One of the most effective ways to achieve flame retardancy in buildings, which usually lose their load-bearing capacity in a fire and cause casualties and economic losses, is to improve the high-temperature resistance of the material itself. Cement matrix composites are commonly used as fire-resistant materials for buildings. However, the use of ordinary silicate cement has serious limitations, the most important of which is its tendency to crack and lose strength rapidly at high temperatures [2]. In addition, high-strength concrete has a dense microstructure and good durability, but when encountering fire and high temperatures, the dense microstructure can lead to serious bursting of high-strength concrete [3,4], causing serious damage to human life and property. Therefore, it is essential to study the high-temperature resistance of other cement-based fireproofing materials.

Potassium magnesium phosphate cement (MKPC) is a new type of cementitious material made of potassium dihydrogen phosphate (KH_2_PO_4_), heavy-burning magnesium oxide (MgO), and retarder in a certain ratio [5,6,7]. Its main product is guano stone (MgKPO_4_-6H_2_O), which has the characteristics of rapid setting and high early strength and is usually used for the rapid repair works of concrete structures [8,9,10,11,12]. Compared with ordinary Portland cement, MKPC has good bonding properties and high-temperature resistance [13,14,15,16,17] and has good prospects for application in the field of fire protection of building structures.

Experimental studies showed that MKPC has good physical and mechanical properties, and the structural fireproof coatings based on MKPC have excellent fire retardant properties. Zheng et al. [18] studied the effect of compounding fly ash and silica fume on the water resistance performance of MKPC. It was found that the density and late compressive strength of MKPC mixed with fly ash and silica fume were higher than those of MKPC without fly ash and silica fume under both air and water curing. Fang et al. [19] studied the fire resistance performance of MKPC doped with expanded perlite (EP), which included loss by weight (LOW), generated cracks, and microstructural changes. The results revealed the feasibility of the MKPC-EP composite as a coating applied to protect the substrate from fire. Vijin et al. [20] studied the ability of magnesium phosphate cement (MPC) coating to protect steel structures and found that no MPC coating peeled off during 45 min of burning under the flame. Dai et al. [21] prepared a magnesium phosphate cement coating by mixing it with expanded vermiculite to study the physical and mechanical properties and fire performance of MPC coating. It was found that when the MPC coating thickness reached 10 mm, its fire performance met the fire resistance requirements of steel structures. At present, there are relatively more studies on fireproof coatings based on magnesium phosphate cement, but most of them only study its fireproof performance under flame contact for less than 1 h. However, for the study of MKPC mortar, there is no clear optimal raw material ratio, and there are few reports on the study of the performance and mechanical properties of MKPC mortar at high temperatures based on the compounding of silica fume and fly ash.

The purpose of this study is to consider the mechanical properties of MKPC with different M:P under fly ash and silica fume compounding conditions. Based on the better M:P, the high-temperature resistance of MKPC under the compounding of fly ash and silica fume was studied. The appearance and compressive strength of MKPC after high temperatures with different fly ash and silica fume admixtures were analyzed, and the variation of the internal temperature of MKPC composite laminate with optimal ratio was evaluated with time.

## 2. Experimental Design

### 2.1. Raw Materials

The raw materials used in this work are provided by Chinese manufacturers. The purity of MgO powder (1200 mesh) is greater than 95%; the purity of potassium dihydrogen phosphate (KH_2_PO_4_) is 99%, with white fine crystals. Retarding agent is borax, the content is more than 95%. In addition, fly ash and silica fume are industrial grades, with a particle size of 1200–1250 mesh. The particle size of quartz sand is 10–40 mesh. The chemical composition of magnesium oxide, fly ash, and silica fume is shown in Table 1.

### 2.2. Preparation of Specimens and Test Methods

The test block preparation process was as follows: first, calculate the amount of material according to the ratio, weigh the exact value of each material; then put the weighed material into the mixer for 1 min to dry mix, mix it well, add water and mix slowly for 30 s, then mix quickly for 2 min to get MKPC mortar; finally, pour the slurry into the 70.7 mm × 70.7 mm × 70.7 mm and 40 mm × 40 mm × 160 mm molds. Then a layer of maintenance film was laid on top of the test block, and the mold was removed promptly after 1 h and naturally maintained until the experimental age. According to existing studies [6,22,23], the raw material mass ratios M(MgO): M(KH_2_PO_4_) (M:P) were chosen to be 2:1, 3:1, and 4:1, respectively, and the fly ash (FA) was blended at 5%, 10%, and 15%, respectively. In addition, silica fume (SF) was added to control the doping material to 20% of the total cementitious material, and the test fixed water-cement ratio (W/C) was 0.18 and the sand-cement ratio was 0.6. The test formulation is shown in Table 2.

### 2.3. Experimental Methods

1. Setting time

The test was conducted according to the method specified in the Chinese specification “Test method for water consumption, Setting time and stability of standard consistency of cement” (GB/T1346-2011). Because the difference between the initial setting time and the final setting time is short, only the initial setting time is tested as the setting time of MKPC cement mortar.

2. Compressive and flexural strength test

Referring to the Chinese specification “Test method of cementitious sand strength (ISO method)” (GB/T17671-1999), the sizes of compressive and flexural specimens are 70.7 mm × 70.7 mm × 70.7 mm and 40 mm × 40 mm × 160 mm, respectively, 3 specimens are tested at each age and the average value is taken as its strength value.

3. High-temperature mechanical property test

**Figure 1:**
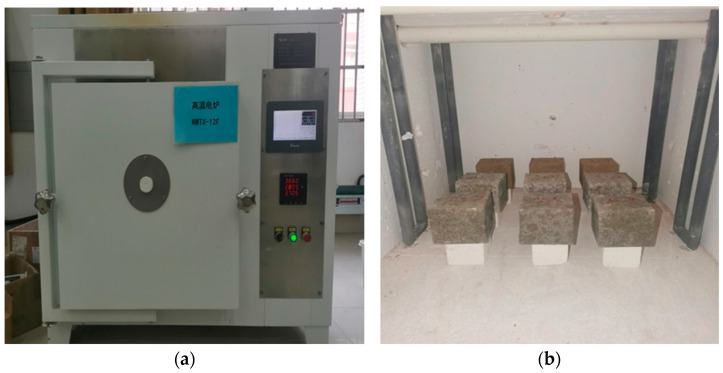
High-temperature electric furnace and test pieces in the furnace: (**a**) High-temperature electric furnace, (**b**) Test piece.

This work mainly carries out the test of the compressive strength of specimens after high temperatures. As shown in Figure 1, the high-temperature furnace is Navit series high-temperature electric furnace, model: NWTX-12F. 28 d age cubic mortar specimens are used for the test blocks. According to tests (1) and (2), the optimum M:P was determined to study the compressive strength of cubic mortar specimens after 3 h at high temperatures of 400 °C, 600 °C and 800 °C under double admixture of fly ash and silica fume.

In order to avoid the mortar test block due to high moisture content, causing it to explode in the high-temperature furnace, before the high-temperature test, the test block was placed in the high-temperature electric furnace using 110 °C drying for 24 h, and then removed and cooled to room temperature. Then, the drying of the test block placed in the high-temperature real electric furnace set the high-temperature furnace heating rate of 5 °C/min. Furthermore, for high-temperature furnace temperature to reach the set temperature, remain at constant temperature for 3 h. After 3 h constant high temperature, the same rate of 5 °C/min cooling to 200 °C, then slowly open the door, so that the temperature in the high-temperature furnace is down to room temperature. Finally, the test block was taken out again and placed in the natural environment of the laboratory, and its compressive strength was measured after three days.

4. High-temperature resistance test of potassium magnesium phosphate laminate

**Figure 2:**
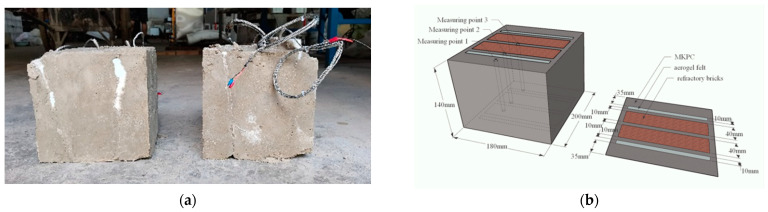
Potassium magnesium phosphate laminate (unit: mm): (**a**) Physical picture, (**b**) Schematic.

According to the mechanical property test before and after high temperature, the optimal ratio among them was selected to prepare potassium magnesium phosphate laminate. Firstly, the aerogel mat and refractory bricks were placed inside the template to fix their positions, and then the prepared potassium magnesium phosphate cement mortar was poured into the positioned template, during which the temperature sensor was inserted into the corresponding position. In addition, after demolding, it was placed in the laboratory for 14 days in a natural environment for a fire test. The heating regime of the test was the same as that of the high-temperature mechanical properties and is not described here. The temperature change was recorded every 3 min during the heating period and every 5 min during the constant temperature period. The temperature sensor model is K-type PT100, and the schematic diagram of the test piece is shown in Figure 2.

## 3. Results and Analysis

### 3.1. Effect of Setting Time

**Figure 3:**
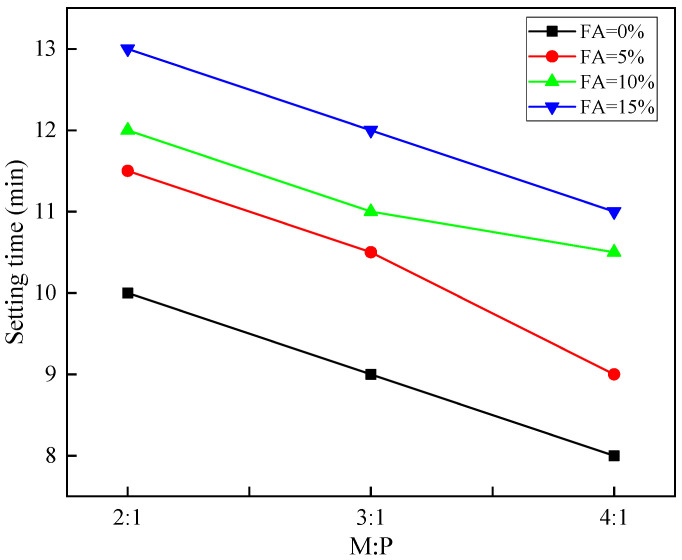
Setting time under different M:P.

The setting time of MKPC cement mortar with different raw material ratios is shown in Table 2. As can be seen from Figure 3, the setting time of MKPC slurry decreases with the increase of M:P, but the overall variation range is not large, between 8 and 13 min. In addition, the growth rate of the setting time of MKPC slurry is not much different under the same amount of fly ash and silica fume admixture. When the M/P is larger, the setting time of cement mortar is shorter. The greater the MgO content, the shorter the setting time of MKPC, which indicates that the setting time is related to the concentration of Mg^2+^ in the cementitious material. The greater the MgO content, the faster its dissolution rate, the more favorable the hydration reaction, the more Mg^2+^ enters the cementitious, and the shorter the setting time [24,25].

**Figure 4:**
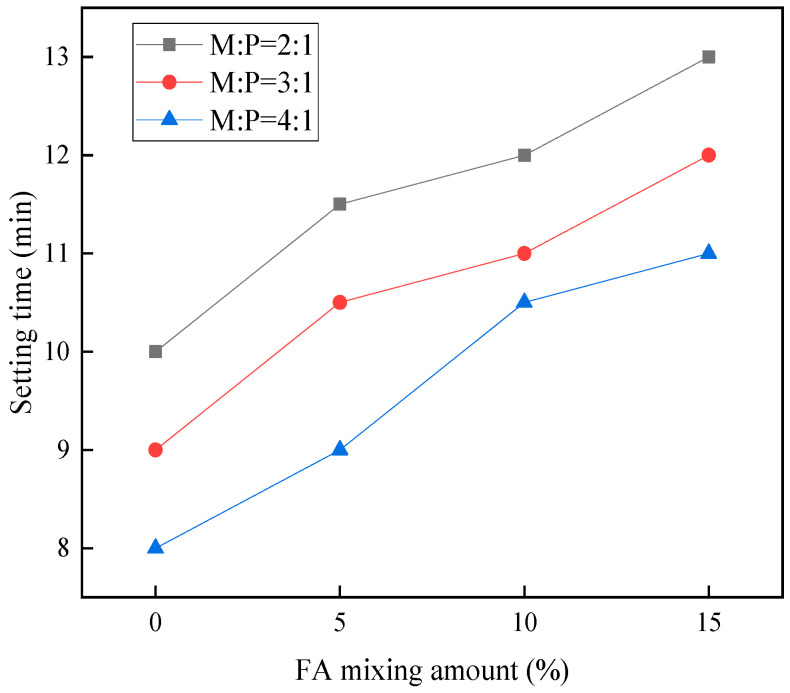
Setting time under different fly ash dosing.

It can be learned from Figure 4 that when the content of M:P is kept constant and the percentage content of fly ash is increased, the setting time of cement mortar grows, but the effect of extending the setting time of the MKPC slurry is limited. The reason for this is that fly ash replaces the reaction of MgO with potassium dihydrogen phosphate, which reduces the amount of MgO that reacts, thus prolonging the setting time of MKPC cement mortar [26]. Moreover, fly ash and silica fume can delay the setting time of MKPC to a certain extent when the amount of fly ash and silica fume is 20% of the total cementitious material. The reason for this is that the incorporation of fly ash and silica fume replaces part of MgO, which reduces the concentration of Mg^2+^ in the slurry and thus prolongs the setting time. In addition, at the same M:P, the setting time of MKPC slurry decreased with the decrease of fly ash admixture and the increase of silica fume admixture, but the decrease was limited. This is similar to the phenomenon observed in a previous study [18].

### 3.2. Effect of Compressive Strength

The compressive strength of potassium magnesium phosphate cement mortar cubic specimens is shown in Table 3. It can be seen from the table that the compressive strength of the specimen with an M:P of 2:1 at the age of 28 d is the largest, and the maximum can reach 55.63 MPa. Furthermore, the strength of the specimen without fly ash and silica fume is slightly greater than that with the addition of fly ash and silica fume, which is because the incorporation of fly ash and silica fume reduces the amount of guano stone (MgKPO_4_·6H_2_O) generated [27]. It can also be found from Table 3 that the compressive strength of MKPC mortar at 1 d and 7 d with the same M:P decreases with the increase of fly ash admixture and increases with the increase of silica fume admixture. The law of 28 d age is different, the specimens with M:P of 3:1 and 4:1 show that the compressive strength of specimens with fly ash and silica fume admixture is greater than the compressive strength of net mortar. The reason for this is that when M:P is small, MgO reacts with KH_2_PO_4_ to produce more products, which is the main provider of MKPC strength. When M:P is greater than 2:1, the products generated by the reaction of MgO with KH_2_PO_4_ are reduced, while fly ash and silica fume act as cementitious materials to provide the micro aggregate effect, which increases the strength of MKPC [28,29]. In addition, the compressive strength of the specimens shows obvious early strength performance when M:P is 2:1. The early strength performance of MKPC is relatively reduced when M:P is 3:1. When M:P is 4:1, MKPC basically no longer has early strength performance, but the compressive strength at 28 d is substantially increased.

**Figure 5:**
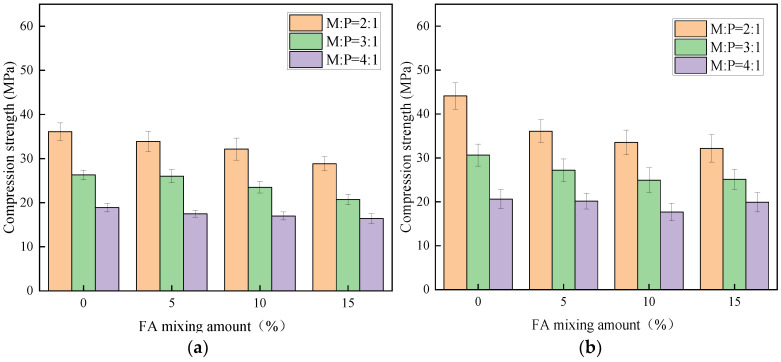

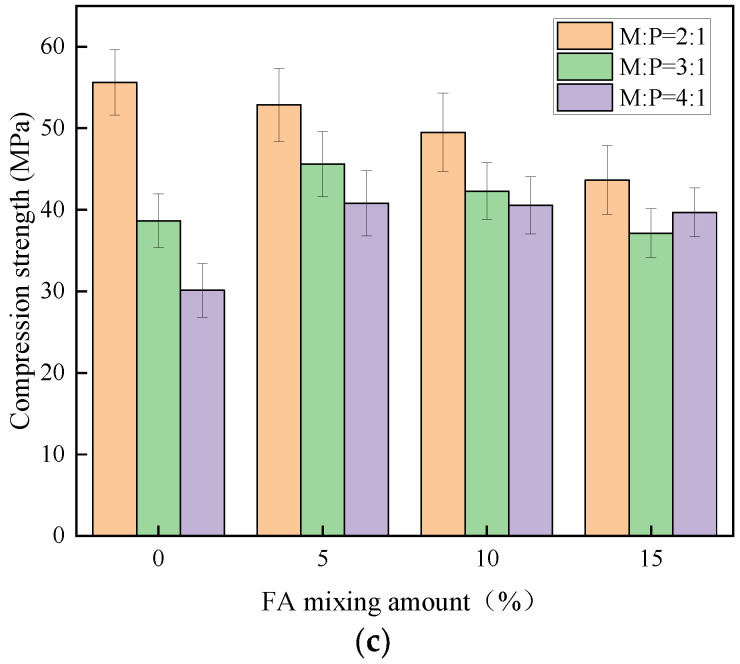
Compressive strength of potassium magnesium phosphate cement at different ages (unit: MPa): (**a**) Compressive strength of 1 d, (**b**) Compressive strength of 7 d, (**c**) Compressive strength of 28 d.

As can be seen from Figure 5, the 1 d compressive strength of MKPC with M:P of 2:1 and 3:1 reaches more than 60% of the 28 d compressive strength with early strength characteristics, while the early strength characteristics of M:P of 4:1 are not obvious and the strength develops relatively slowly with time. Figure 5a–c represent the compressive strengths of cubic specimens at 1 d, 7 d, and 28 d ages, respectively. Figure 5a shows that the strength of M:P = 3:1 decreased by 27.2%, 23.2%, 27%, and 28.1% relative to 2:1 under the conditions of 0%, 5%, 10%, and 15% of FA admixture, respectively, while the strength of M:P = 4:1 decreased more than that of 2:1, respectively. Figure 5a,b show that the incorporation of fly ash decreases the compressive strength of the specimens for the same M:P, the same conclusion was reached in reference [30]. Figure 5c shows that the compressive strength of specimens with M:P of 2:1 decreases with the decrease of silica fume (SF) admixture, while the specimens with M:P of 3:1 and 4:1 show a trend of first increase and then decrease. The reason is that, with the increase of M:P, the production of guano stone decreases more, and the micro-aggregate filling effect of silica fume is reflected to a greater extent at this time, which also indicates that silica fume can enhance the compactness of the cement matrix, thus achieving the effect of increasing the strength [31].

By comparison, it was found that the compressive strength of MKPC was influenced by M:P, and the compressive strength was maximum when M:P was 2:1. When M:P was greater than 2:1, the compressive strength of the specimens was significantly reduced.

### 3.3. Effect of Flexural Strength

**Figure 6:**
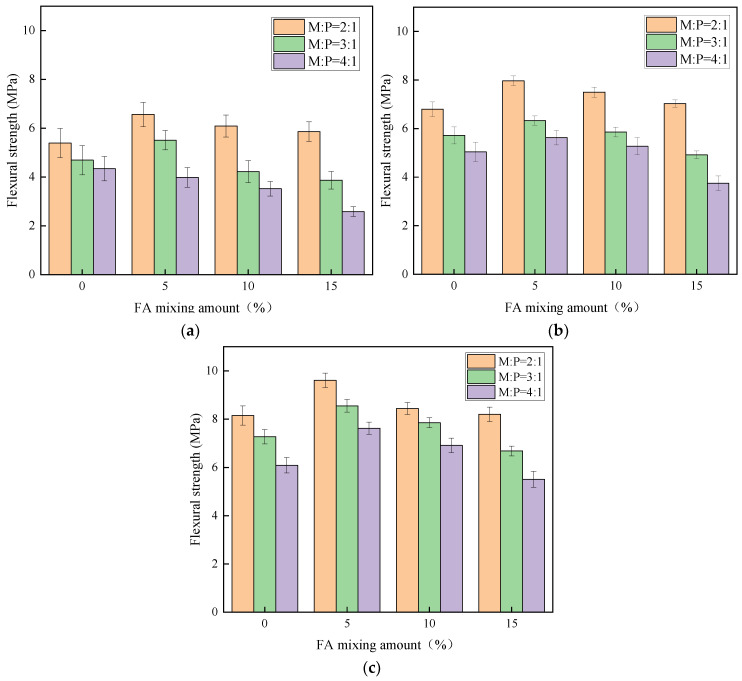
Flexural strength of potassium magnesium phosphate cement at different ages (unit: MPa): (**a**) Flexural strength of 1 d, (**b**) Flexural strength of 7 d, (**c**) Flexural strength of 28 d.

From Table 3, the maximum flexural strength of MKPC is 9.61 MPa. Unlike the compressive strength, the flexural strength of MKPC with 5% fly ash and 15% silica fume is stronger than other ratios under the same M:P. This is mainly because silica fume has the micro-aggregate filling effect, which is beneficial to improve the compactness of potassium magnesium phosphate cement mortar [29]. Figure 6 shows the flexural strength of MKPC at different ages, when M:P is the same, the flexural strength shows a decreasing trend with the increase of FA doping. When the amount of FA and SF is the same, the larger the M:P is, the smaller the flexural strength is, and its early strength is influenced by M:P, and the specimen at 28 d age is less influenced by M:P. The main reason is that a certain amount of silica fume can give full play to the micro-aggregate filling effect and improve the particle gradation of MKPC mortar, while the silica fume particles can be used as nuclei to promote the development of hydration of potassium magnesium phosphate cement, which is beneficial to the development of flexural strength of potassium magnesium phosphate cement with the growth of age [32].

It can also be seen from Figure 6 that the flexural strength of MKPC with M:P of 2:1 is significantly higher than that of the specimens with other ratios, and the incorporation of silica fume can effectively improve the flexural strength. Under the same age, the flexural strength of MKPC mortar shows an increasing trend with the increase of silica fume admixture. In addition, when the M:P is 2:1, the flexural strength of the specimens shows obvious early strength performance, and its 1 d flexural strength can reach more than 65% of the 28 d flexural strength. When the M:P is 3:1, the early strength performance of MKPC is relatively reduced. When the M:P is 4:1, the early flexural strength of MKPC is too low, but the 28 d flexural strength is greatly improved. On the other hand, the flexural strength of MKPC with silica fume is significantly better than that of net paste when M:P is 2:1. MKPC with M:P of 3:1 and 4:1 can significantly improve the flexural strength of MKPC when the silica fume admixture is 15%. When the silica fume admixture is less than 10%, the flexural strength of the specimens basically no longer increases compared with the MKPC net paste. This is because when M:P is small, MgO reacts with KH_2_PO_4_ to generate more products, which is the main provider of the strength of MKPC [24], and the admixture of silica fume plays the role of filling the pores of the matrix. When M:P is greater than 2:1, the products generated by the reaction between MgO and KH_2_PO_4_ are reduced and more unreacted MgO remains [25,33], which leads to a decrease in the strength of MKPC.

### 3.4. Compressive Strength after High Temperature

**Figure 7:**
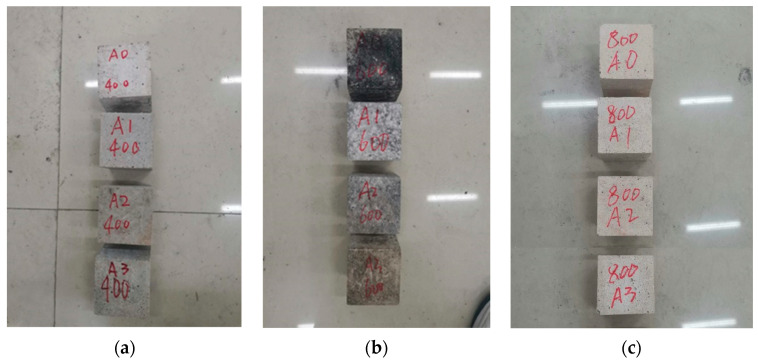
Experimental specimens after high-temperature overheating: (**a**) 400 °C, (**b**) 600 °C, (**c**) 800 °C.

Through the mechanical properties test, the optimal M:P was determined to be 2:1. Therefore, cubic mortar specimens with an age of 28 d and an M:P of 2:1 were used for the high-temperature experiments. Figure 7 shows the experimental specimens after high-temperature tempering at 400 °C, 600 °C, and 800 °C. By comparison, it was found that the surface of the specimens after 400 °C high temperature was from white to gray with the decrease of silica fume dosing, and all of them were accompanied by the appearance of micro-cracks. In addition, a large number of curved cracks appeared on the surface of the A0 specimen; the cracks on the surface of the A1 specimen were not obvious and relatively small; there were no obvious cracks on the surface of the A2 specimen; there were a large number of small pores on the surface of the A3 specimen without obvious cracks. After 600 °C high temperature, the surface of the A0 specimen showed black color with obvious long cracks; the surface of the A1 specimen was light gray and the cracks were net-like; the surface color of the A2 specimen was deepened and black spots appeared with more obvious cracks; the surface color of the A3 specimen showed large black spots with no obvious cracks. Furthermore, the color of the 800 °C high-temperature specimen was lighter and had more cracks compared with the previous two temperature specimens. At the same temperature, the color change was caused by the doping of fly ash and silica fume. Cracks were generated because of the temperature difference between the internal and surface temperatures of the specimen, and the existence of closed pores inside MKPC. When the gas inside the pores was heated and expanded, it caused tensile stresses inside the concrete, which resulted in cracks on the surface of the specimen [34].

It was found that the surface color of the specimen was mainly related to the temperature through the high-temperature test, and the correlation with the amount of fly ash and silica fume doping was not significant. With the increase in temperature, the surface color of the specimens changed from dark to light. In addition, the amount of fly ash and silica fume doping did not have a regular effect on the distribution of cracks on the surface of the specimen but had a significant effect on the number of cracks. Under the same temperature, the number of cracks on the surface of the specimen gradually decreased with the increase of silica fume dosing, and the number of surface pores also gradually became smaller. On the contrary, the number of cracks and pores on the surface of the net mortar specimens was more than that of the specimens mixed with silica fume. This is due to the high content of guanoite (MgKPO_4_-6H_2_O) in the net slurry specimens, which is thermally decomposed at a high temperature [35] and the loss of crystalline water, resulting in the decrease of strength and the higher number of surface cracks in the specimens. In contrast, the incorporation of silica fume reduced the amount of guanoite production, while increasing the denseness of the matrix. A study [36] conducted high-temperature tests by adding different amounts of silica fume instead of MgO and found that the participation of 10% silica fume significantly improved the high-temperature resistance of MKPC, which is similar to the findings in this paper.

**Figure 8:**
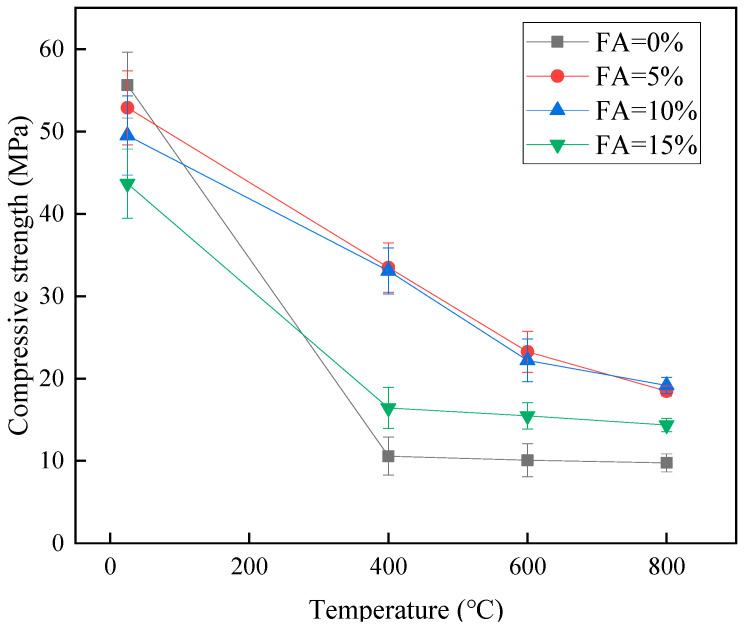
Compressive strength of 28 d specimens after high temperature (unit: MPa).

Table 4 shows the compressive strength values of MKPC cubic specimens after high-temperature overheating, and the compressive strengths were determined at high temperatures of 400 °C, 600 °C, and 800 °C, respectively. As can be seen from Table 4, the compressive strengths of the specimens with 0% and 5% silica fume doping were only 19.0% and 37.6% of the 28 d compressive strength after 400 °C high temperature. However, the compressive strengths of MKPC specimens with 15% and 10% silica fume doping were 63.3% and 66.8% of the 28 d compressive strength after 400 °C, respectively. With the increase of temperature to 800 °C, the MKPC with 15% or 10% silica fume doping still had higher compressive strength. This indicates that silica fume can significantly improve the high-temperature resistance of MKPC. Figure 8 shows the change of compressive strength at different high temperatures, and it is found that high temperature has a greater effect on the compressive strength of MKPC, and the compressive strength becomes smaller when the temperature increases. As can be seen from the figure, when the temperature increases to 400 °C, the strength of the specimens with 0% and 5% silica fume doping decreases sharply, but as the temperature increases to 600 °C and 800 °C, the strength no longer changes; unlike it, the specimens with 10% and 15% silica fume doping still have high compressive strength after 3 h of constant temperature at 400 °C and constant 600 °C, and the change in compressive strength of MKPC with the increase of temperature t is small, and the difference of compressive strength under the same high temperature is not large. The reason is that the water of crystallization in the guano stone in potassium magnesium phosphate cement is affected by high temperature, which leads to the evaporation of water and the formation of bubbles, causing the internal structure to be incompact, thus reducing the compressive strength [37]. According to the results of Vijan et al. [17], the decomposition and dehydration of guano stone during the burning of the fireproof coating of phosphate cement (MPC) under flame is the main reason for the decrease in the mechanical properties of MPC. In contrast, silica fume can increase the compactness of the matrix and is less affected by high temperature, so the right amount of silica fume can improve the high-temperature resistance of MKPC.

### 3.5. High-Temperature Resistance of Laminate

**Figure 9:**
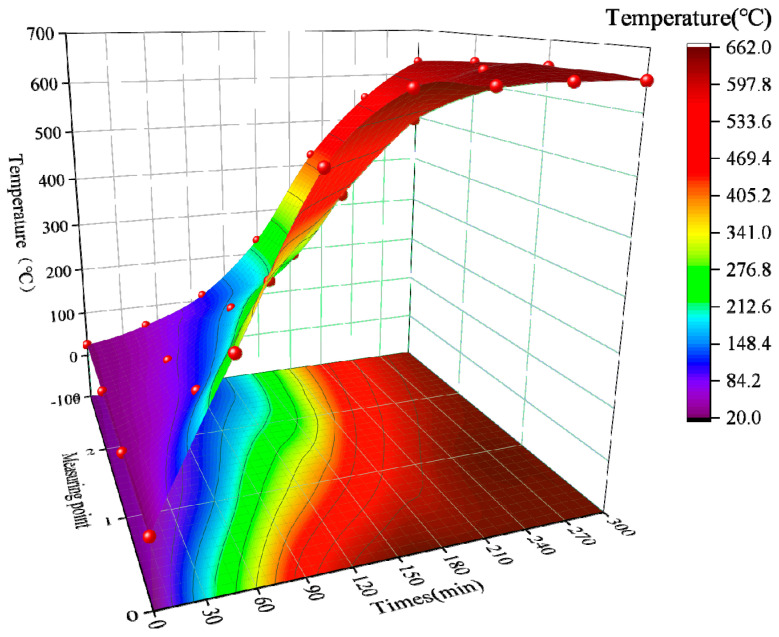
Surface plot of the temperature variation of measurement points with time.

As shown in Figure 9, points 0, 1, 2, and 3 are the high-temperature furnace temperature and its three measurement points, respectively, and the color gradually changes from light to dark as the temperature increases. During the period of 0–60 min, the temperature of the three measurement points changed slowly; during 60–120 min, the temperature of measurement point 1 increased from 147 °C to 382 °C, and the temperature increase rate crossed several temperature gradients, but the measurement point 2 and measurement point 3 were still in the slow heating state. At the end of the temperature rise, the temperature inside the furnace was dark red 650 °C, with a difference of 268 °C from measurement point 1, which shows that the heat insulation of potassium magnesium phosphate cement is good, while measurement point 2 and measurement point 3 reached green-blue 201 °C and blue 134 °C, respectively. With constant temperature 120–210 min period measurement point 1 warming rapidly, from 382 °C to 592 °C, the subsequent measurement point 1 warming becomes slower, until 270 min to 650 °C, after that measurement point 1 in 650 °C shows tiny fluctuations. Measurement point 2 and measurement point 3 in the constant temperature 120–270 min stage maintain a faster heating rate, after which the temperature change tends to level off. In addition, at the end of the constant temperature, measurement point 2 and measurement point 3 reached 644 °C and 628 °C, respectively. From the time-temperature surface plot in Figure 9, it can be seen that the temperature difference between the adjacent measurement points 0~4 gradually becomes smaller in the same heating or constant temperature time, which indicates that the thermal insulation performance of potassium magnesium phosphate cement is better than that of aerogel mat and refractory brick.

It has been shown [38,39] that MKPC starts to lose crystal water at about 60 °C and the decomposition is completed at about 250 °C. From the experimental phenomena, it was found that the temperature rise rate is slow until the internal temperature of the MKPC layer rises to 260 °C. However, the rate of rise increased significantly after the internal temperature of MKPC exceeded 260 °C. Apparently, this is related to the degree of guanoite dewatering in the MKPC matrix. Before the guano stone was completely dehydrated, MKPC maintained a high strength and high-temperature resistance, and when MKPC was completely dehydrated, its high-temperature resistance decreased.

### 3.6. Microstructure Analysis

**Figure 10:**
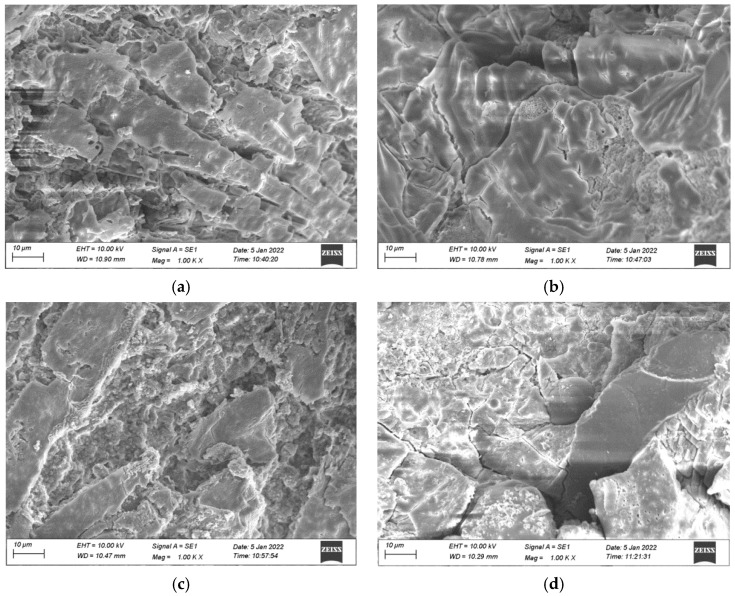
The microscopic morphology of MKPC at the age of 28 days with M:P = 2:1. (**a**) A0; (**b**) A1; (**c**) A2; (**d**) A3.

The fracture surface of the crushed cubic specimens at the age of 28 d was gold blasted, and the peritectic morphology of the MKPC mortar section was observed by EVO-MA/LS scanning electron microscopy (SEM) as shown in Figure 10. In this work, the experimental temperature was 25 °C.

SEM analysis was performed on specimens A0, A1, A2, and A3, and the results are shown in Figure 10. The specimen in Figure 10a has a tight internal structure, and a large number of guttered massive crystals can be seen lapping each other, and the generated product guanoite (MgKPO_4_·6H_2_O) provides the strength of the specimen. Figure 10b shows that the guanoite crystals produced by the hydration of MKPC lap each other and encapsulate the unreacted MgO, forming a dense structure with a smooth surface. In Figure 10c, with the increase of fly ash, unreacted MgO attached to the surface of the structure with smaller voids and increased flocculation, which wrapped around the guanoite and made the structure dense. Cracks appear in Figure 10d, with many exposed fly ash and MgO attached to the surface, and the overall denseness is reduced. From the microstructure diagram of MKPC, it can be seen that with the increase of fly ash admixture and the decrease of silica fume admixture, there are more and more pores and cracks, and the decrease of colloidal products and compactness leads to the decrease of compressive strength and flexural strength of MKPC mortar.

SEM analysis showed that the denseness of MKPC improved with fewer cracks and defects when fly ash and silica fume were used in combination [18]. However, under the dual admixture of silica fume and fly ash, the increase of fly ash admixture caused more flocculation inside the MKPC matrix, which led to a decrease in its strength. According to Ding et al. [40], in addition to crystalline guanoite, amorphous guanoite is also formed in the MKPC matrix, with crystalline guanoite the main provider of the strength of the MKPC matrix. A large amount of crystalline guanoite can be found in the SEM microscopic morphology of A0 and A1, and the SEM photograph of A2 shows significantly less guanoite and more flocculent material. Although there is no obvious flocculent material in A3, the number of matrix cracks is very obvious. It can be seen that guanoite is the main provider of the strength of the MKPC matrix, and silica fume can increase its denseness, thus improving its strength.

## 4. Conclusions

From this work, the following conclusions have been reached:

(1) The quality ratio of raw materials has a direct influence on the mechanical properties of MKPC, among which M:P has a greater influence on the strength of MKPC.

(2) The admixture of fly ash and silica fume reduces the early compressive strength of MKPC but has little effect on the mechanical properties at 28 d. When the M:P is 2:1, fly ash doping is 5% and silica fume doping is 15%, the mechanical properties and high-temperature resistance of MKPC reach the optimum.

(3) Silica fume can significantly improve the high-temperature resistance of MKPC. After 3 h of high temperature, the compressive strength of potassium magnesium phosphate cement mortar with silica fume dosing of 0% and 5% decreased significantly, but the effect was less for MKPC with silica fume dosing of 10% or 15%.

(4) Analysis of the cross-sectional micro-morphology of MKPC mortar specimens with M:P = 2:1 observed by SEM, silica fume can increase the compactness of MKPC mortar, thus increasing its strength.

(5) The heat insulation performance of MKPC laminate is better under the optimal ratio, the temperature inside the laminate increases slowly with time, and the temperature change of MKPC is better than that of aerogel mat and refractory brick at the same time.

## Figures and Tables

**Table 1:** Chemical composition of magnesium oxide, fly ash, and silica fume (%).

Raw Materials	MgO	SiO_2_	CaO	Fe_2_O_3_	Al_2_O_3_	NaOH	SO_3_	C
Magnesium oxide	90.72	4.61	2.24	1.48	0.41	0.54	-	-
Fly ash (FA)	1.72	60.7	0.6	4.67	30.71	-	1.6	-
Silica fume (SF)	-	96.7	-	1.37	-	-	-	1.93

**Table 2:** The mass ratio of raw materials.

Symbol	M:P	FA	SF	B/M	W/C	Setting Time (min)
A0	2:1	0%	0%	0.05	0.18	10
A1	2:1	5%	15%	0.05	0.18	11.5
A2	2:1	10%	10%	0.05	0.18	12
A3	2:1	15%	5%	0.05	0.18	13
B0	3:1	0%	0%	0.05	0.18	9
B1	3:1	5%	15%	0.05	0.18	10.5
B2	3:1	10%	10%	0.05	0.18	11
B3	3:1	15%	5%	0.05	0.18	12
C0	4:1	0%	0%	0.05	0.18	8
C1	4:1	5%	15%	0.05	0.18	9
C2	4:1	10%	10%	0.05	0.18	10.5
C3	4:1	15%	5%	0.05	0.18	11

Note: M:P indicates the mass ratio of magnesium oxide to potassium dihydrogen phosphate, B/M indicates the mass ratio of borax to magnesium oxide, and W/C indicates the water-ash ratio.

**Table 3:** Compressive and flexural strengths of mortar test blocks at 1 d, 7 d, and 28 d. (Unit: MPa).

Symbol	1 d		7 d		28 d	
	Compressive Strength	Flexural Strength	Compressive Strength	Flexural Strength	Compressive Strength	Flexural Strength
A0	36.10	5.39	44.12	6.80	55.63	8.15
A1	33.88	6.56	36.06	7.97	52.87	9.61
A2	32.16	6.09	33.53	7.50	49.51	8.44
A3	28.82	5.86	32.17	7.03	43.66	8.20
B0	26.29	4.69	30.64	5.72	38.65	7.27
B1	26.02	5.51	27.18	6.33	45.61	8.55
B2	23.49	4.22	24.92	5.86	42.28	7.85
B3	20.71	3.87	25.12	4.92	37.14	6.68
C0	18.88	4.34	20.60	5.04	30.12	6.09
C1	17.47	3.98	20.16	5.63	40.79	7.62
C2	16.98	3.52	17.66	5.27	40.56	6.91
C3	16.39	2.58	19.90	3.75	39.68	5.51

**Table 4:** Compressive strength of 28 d specimens with M:P of 2:1 after high temperature overheating (unit: MPa).

Symbol	25 °C	400 °C	600 °C	800 °C
A0	55.63	10.57	10.08	9.76
A1	52.87	33.48	23.25	18.47
A2	49.51	33.07	22.20	19.16
A3	43.66	16.42	15.46	14.36

## Data Availability

The data used to support this study are available from the corresponding author upon request.
